# Anti-Inflammatory Activity Comparison among Scropoliosides—Catalpol Derivatives with 6-*O*-Substituted Cinnamyl Moieties

**DOI:** 10.3390/molecules201119659

**Published:** 2015-11-03

**Authors:** Tiantian Zhu, Liuqiang Zhang, Shuang Ling, Fei Qian, Yiming Li, Jin-Wen Xu

**Affiliations:** 1 Murad Research Institute for Modernized Chinese Medicine, Shanghai University of Traditional Chinese Medicine, Shanghai 201203, China; sweet-0801@hotmail.com (T.Z.); sarah_ling@126.com (S.L.); qianfei0517@126.com (F.Q.); 2 School of Pharmacy, Shanghai University of Traditional Chinese Medicine, Shanghai 201203, China; 04100217@163.com

**Keywords:** scropoliosides, 6-*O*-substituted cinnamyl moiety, antiinflammatory effect, NF-κB, cytokines

## Abstract

We have previously shown that scropolioside B has higher anti-inflammatory activity than catalpol does after the inhibition of nuclear factor (NF)-κB activity and IL-1β expression, maturation, and secretion. Various scropoliosides were extracted, isolated, and purified from *Scrophularia dentata* Royle ex Benth. We then compared their anti-inflammatory activities against LPS-induced NF-κB activity, cytokines mRNA expression, IL-1β secretion, and cyclooxygenase-2 activity. The inhibitory effects of the scropoliosides varied depending on whether the 6-*O*-substituted cinnamyl moiety was linked to C′′ 2-OH, C′′3-OH, or C′′4-OH, and on the number of moieties linked, which is closely related to the enhancement of antiinflammatory activity. Among these compounds, scropolioside B had the strongest antiinflammatory effects.

## 1. Introduction

**Figure 1:**
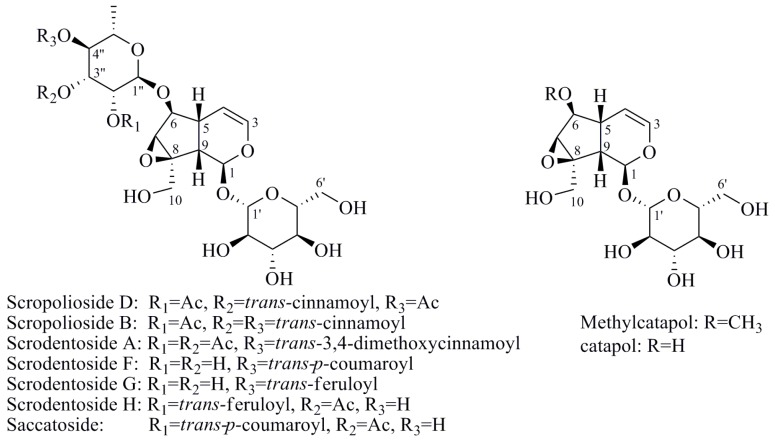
Chemical structures of scropoliosides and catalpol.

Iridoids are a class of secondary metabolites found in a wide variety of various plants, such as Scrophulariaceae, Loganiaceae, Gentianaceae, Rubiaceae, Verbenaceae, and Oleaceae, *etc*. Iridoids have neuroprotective, antiinflammatory, immunomodulatory, hepatoprotective, cardioprotective, anticancer, antioxidant, antimicrobial, hypoglycemic, hypolipidemic, choleretic, antispasmodic, and purgative properties [1,2,3,4]. Hydrolyzed products of iridoid glycosides, harpagide and harpagoside, exhibited a dose-dependent inhibitory effect on cyclooxygenase-2 (COX-2) activity at 2.5–100 μmol/L [5]. Catalpol and aucubin are two of the most common iridoids and exhibit weak antiinflammatory effects [6]. At 500 μmol/L, catalpol reduced the expression of proinflammatory mediators, such as monocyte chemotactic protein-1 (MCP-1), tumor necrosis factor-α (TNF-α)-inducible NO synthase, and the receptor for advanced glycation endproducts (AGE), and significantly reduced the transcriptional activation of nuclear factor (NF)-κB [7]. Fu *et al.* [8] reported that catalpol inhibited myeloperoxidase activity in lung samples and reduced mouse lung wet-to-dry weight ratio, the amounts of inflammatory cells, TNF-α, IL-6, IL-4, and IL-1β in mouse bronchoalveolar lavage fluid, and the amount of alveolar macrophages in male BALB/c mice. Moreover, catalpol upregulated the production of IL-10 in bronchoalveolar lavage fluid and alveolar macrophages. Recently, we observed that scropolioside B, a catalpol derivative, effectively inhibited IL-1β and other cytokines expression at 50 μmol/L through NF-κB and NLRP3 pathways [9]. However, catalpol did not effectively inhibit NF-κB activity at the same concentration, suggesting that the antiinflammatory effect of scropolioside B is higher than that of catalpol. Bas *et al.* [10] also reported that scropolioside A (100 μmol/L) inhibited the production of PGE_2_, LTB_4_, NO, IL-1β, IL-2, IL-4, TNF-α, and IFN-β; however, it did not affect the production of IL-10. However, because of the diversity of iridoid structure and lack of clarity about its relationship with the activity, we chose eight scropoliosides (Figure 1), namely scrodentoside A, B, and D, 6-*O*-α-l-(4′′-*O*-trans-*p*-coumaroyl) rhamnopyranosylcatalpol (named scropolioside F) [11], 6-*O*-α-l-(4′′-*O*-feruloyl) rhamnopyranosylcatalpol (scropolioside G) [12], 6-*O*-α-l-(2′′-*O*-feruloyl) rhamnopyranosylcatalpol (scropolioside H) [13], saccatoside [14], and 6-*O*-methylcatapol [15], which were isolated from *Scrophularia dentata* Royle ex Benth, and analyzed the effects of their structure-activity relationships on the antiinflammatory effect.

## 2. Results and Discussion

### 2.1. Effect of Iridoid Glycosides on NF-κB Activation

**Figure 2:**
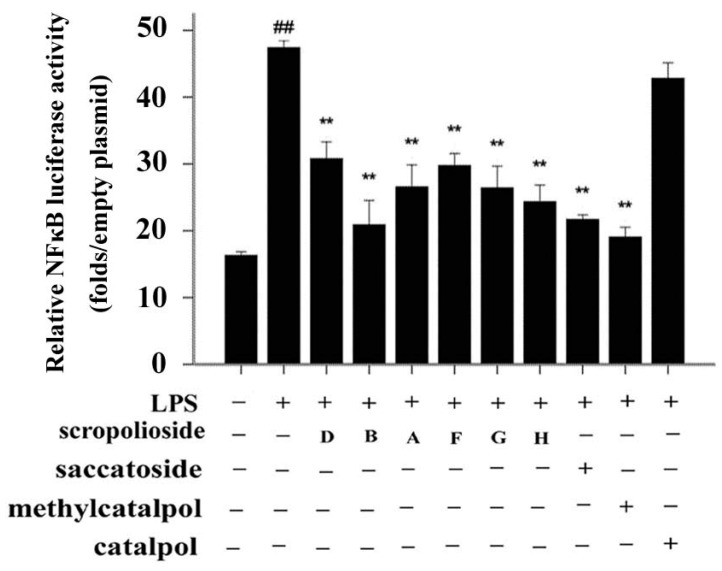
Effects of scropoliosides and catalpol on TNF-α-induced NF-κB activation. Cells were preincubated for 1 h with 50 μmol/L scropoliosides or catalpol and then stimulated with 100 ng/mL of TNF-α for 16 h. The results shown are representative of 3 repeated experiments. Data are expressed as means ± SD. **##** *p* < 0.01 *vs.* the control, ** *p* < 0.01 *vs.* the TNF-α group.

Because all iridoid structures contain a catalpol skeleton (Figure 1), we compared and analyzed the antiinflammatory activities of iridoids and catalpol in HEK293 cells transfected with the luciferase reporter plasmid. To investigate the overall antiinflammatory activity of these monomers, we used a luciferase reporter assay to determine NF-κB activity. After HEK293 cells were transferred with NF-κB or the control plasmid, the cells were incubated with or without the monomer for 1 h and then stimulated with 100 ng/mL of TNF-α. The luciferase activity increased after stimulation with TNF-α. Pretreatment with 50 μmol/L iridoid glycosides, but not catalpol, resulted in 40%–60% inhibitory effect on NF-κB luciferase reporter activity, suggesting that methyl- or glycoside-modified groups at the 6 site increase the ability of the compound to inhibit NF-κB activation (Figure 2).

### 2.2. Cytokine Expression

**Figure 3:**
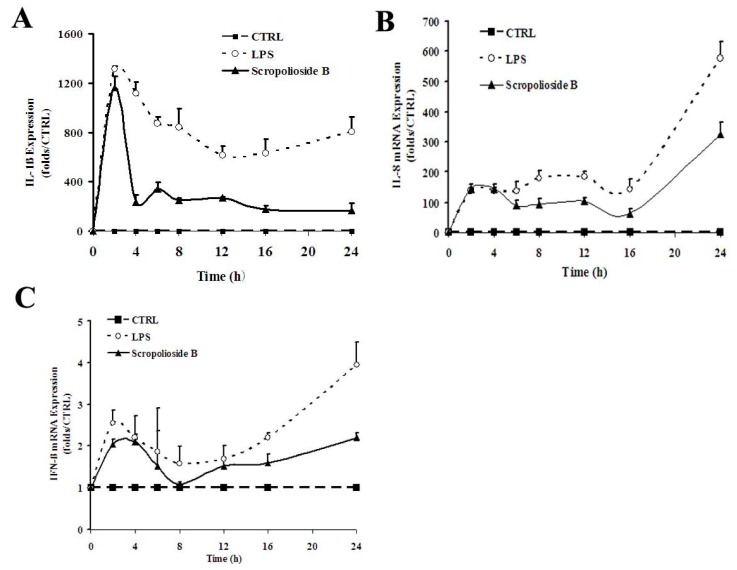
Kinetics of the inhibitory effect of scropolioside B on the expression of IL-1β, IL-8, and IFN-β in LPS-induced THP-1 cells. THP-1 cells were pretreated with 50 μmol/L scropolioside B for 1 h and then stimulated with LPS (1 μg/mL) for another 2, 4, 6, 8, 12, 16, or 24 h. The mRNA expression of IL-1β, IL-8, and IFN-β was measured using real-time RT-PCR. The results shown are representative of three repeated experiments.

**Figure 4:**
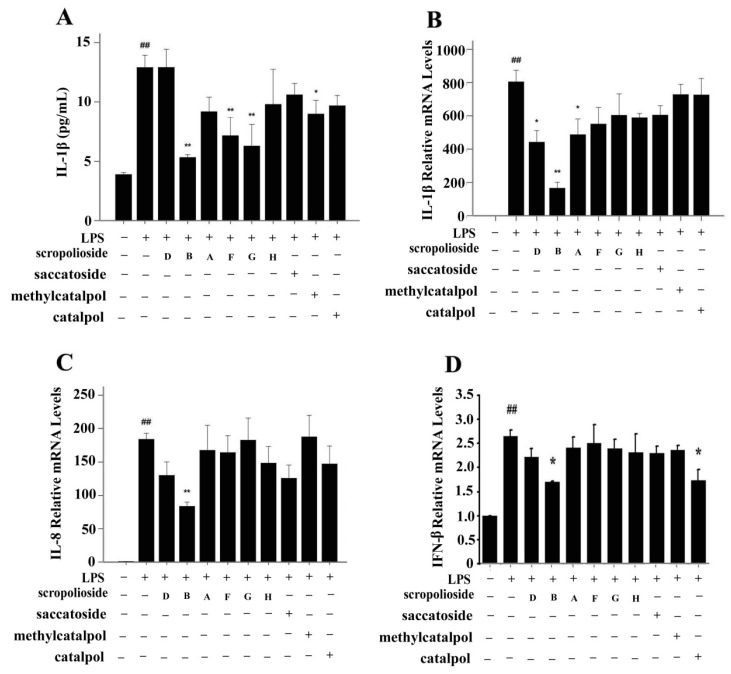
Effect of scropoliosides and catalpol on the expression of IL-1β, IL-8, and IFN-β in LPS-induced THP-1 cells. THP-1 cells were pretreated with 50 μmol/L scropoliosides or catalpol for 1 h and then stimulated with LPS (1 μg/mL) for another 6 or 24 h. (**A**) The secretion of IL-1β in the culture medium from the LPS-induced THP-1 cells was measured using Abcam Human IL-1β ELISA kit; (**B**–**D**) The mRNA expression of IL-1β, IL-8, and IFN-β was measured using real-time RT-PCR. The data represent the mean values of more than three repeated experiments ± SD. **##** *p* < 0.01 *vs.* the vehicle control, ** *p* < 0.01 *vs.* LPS alone, * *p* < 0.05 *vs.* LPS alone.

To understand the effect of these iridoid glycosides on cytokine expression, we selected three cytokines based on signaling pathways induced by LPS/TLR4 and different secretion time, namely IL-1β, IL-8, and IFN-β, and examined the inhibitory effects of the iridoids. In lipopolysaccharides (LPS)-stimulated THP-1 monocytes, IL-1β expression increased rapidly within 2–4 h, and IL-8 and IFN-β expression presented a biphasic pattern with the late peak being higher than the previous peak and scrodentoside B only inhibiting the late peak (Figure 3). In the case of the THP-1 cells treated with various iridoids, scropoliosides B, F, and G and 6-*O*-methylcatapol significantly reduced IL-1β maturation and secretion in the clutured medium of the THP-1 cells (Figure 4A), and only scropoliosides A, B, and D inhibited IL-1β mRNA expression (Figure 4B). Our results showed that scropolioside B, but not other iridoids, inhibited IL-8 mRNA expression (Figure 4C) and that scropolioside B and catalpol reduced IFN-β mRNA expression in the LPS-induced THP-1 cells (Figure 4D).

### 2.3. Activity of Arachidonic-Acid-Metabolizing Enzymes

**Figure 5:**
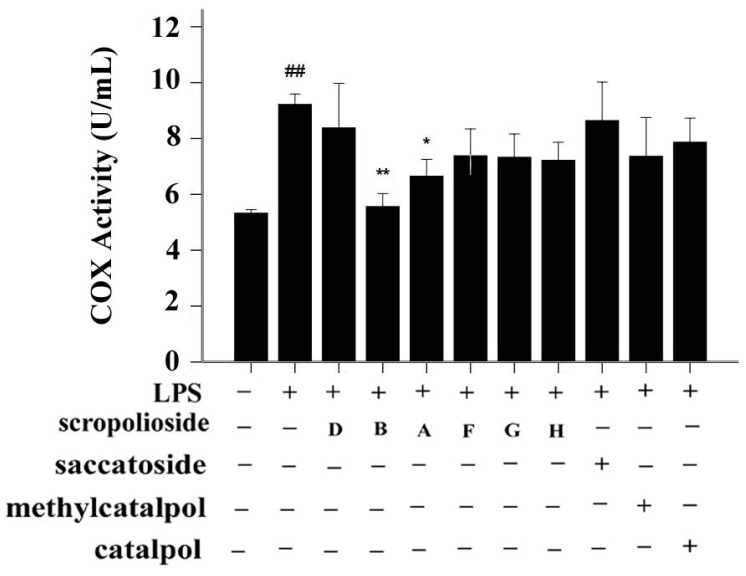
Effects of scropoliosides and catalpol on LPS-induced COX-2 activation. Cells were preincubated for 1 h with 50 μmol/L scropoliosides or catalpol and then stimulated with 1 μg/mL of LPS for 24 h. The results shown are representative of three repeated experiments. Data are expressed as means ± SD. **##** *p* < 0.01 *vs.* the control, ** *p* < 0.01 *vs.* LPS alone, * *p* < 0.05 *vs.* LPS alone.

COX-2, which is induced by inflammatory cytokines, promotes prostaglandin synthesis and mediates reactions involved in pain, inflammation, and fever. To determine whether inflammatory factors induce COX-2 activity, we stimulated THP-1 cells with LPS for 24 h. LPS upregulated COX-2 activity (Figure 5). Pretreatment with scrodentosides A and B inhibited COX-2 activity (Figure 5). We used the 15-LOX inhibitor screening assay kit to analyze inhibitory effects of these iridoids, and found that these iridoids did not inhibit 15-LOX activity (data not shown).

### 2.4. Structure-Activity Relationship of the Eight Catalpol Derivatives and Catalpol

Our results showed that all 6-*O*-substituted catalpol derivatives, whether rhamnopyranosylcatalpol or methyl-modified, exhibited higher inhibitory activities against NF-κB activation than catalpol did, indicating that compounds with low-polarity substituents at the 6-*O* position of catalpol displayed higher NF-κB inhibitory potency (Table 1). Moreover, the cinnamyl group-substituted positions C′′2-OH, C′′3-OH, and C′′4-OH are associated with the antiinflammatory activity against NF-κB activation (Figure 1 and Table 1). For example, compounds with cinnamyl groups linked to C′′2-OH (saccatoside and scrodentoside H) exhibited higher inhibitory effects against NF-κB activation than those with cinnamyl groups lined to C′′3-OH (scrodentosides A and D). Notably, scrodentoside A and D, containing cinnamyl groups linked to C′′3-OH, but not scrodentosides containing cinnamyl groups linked to C′′2-OH or C′′4-OH, effectively prevented IL-1β, IL-8, and IFN-β mRNA expression (Table 1). Conversely, scropoliosides B, F, and G, containing a cinnamyl or feruloyl group at C′′4-OH, effectively blocked IL-1β secretion (Table 1). However, according to the structure–activity relationship, two cinnamyl groups should be included at C′′3-OH and C′′4-OH positions of scropolioside B for its COX-2 inhibitory activity (Table 1).

**Table 1:** Inhibitory ratio of compounds for inflammatory indicators (%, each *n* = 3).

Compounds	Structure	Inhibitiory Ratio of NF-κB Activity	Inhibitiory Ratio of IL-1β mRNA Expression	Inhibitiory Ratio of IL-1β Protein Expression	Inhibitiory Ratio of IL-8 mRNA Expression	Inhibitiory Ratio of IFN-β mRNA Expression	Inhibitiory Ratio of COX-2 Activity
Scropolioside B	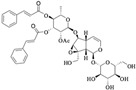	**49.1**	**79.5**	**58.8**	**54.6**	**35.9**	**32.3**
Scropolioside D	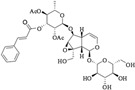	**40.9**	**45.1**	**−0.1**	**29.4**	**16.5**	**−1.9**
Scrodentoside A	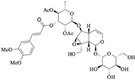	**40.2**	**39.5**	**28.9**	**8.8**	**9.25**	**19.1**
Scrodentoside F	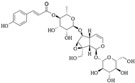	**41.9**	**31.6**	**44.5**	**10.8**	**5.6**	**10.2**
Scrodentoside G	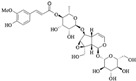	**50.4**	**25**	**51.3**	**0.8**	**10.1**	**10.8**
Scrodentoside H	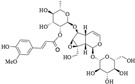	**52.8**	**26.8**	**24.1**	**19.3**	**13.5**	**12.1**
Saccatoside	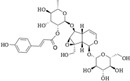	**58.1**	**24.9**	**17.9**	**31.7**	**11.2**	**−5.1**
Methylcatapol	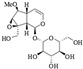	**59.2**	**9.6**	**51.3**	**-2**	**8.9**	**10.4**
Catalpol	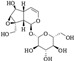	**−2.3**	**9.8**	**25**	**19.9**	**34.7**	**4.2**

### 2.5. Effect of Cinnamyl Moieties in Scropoliosides on Anti-inflammatory Activity

Catalpol, aucubin, and genipin are the basic structural compounds of iridoids, which show weak antiinflammatory activity [16,17,18]. We recently showed that scropolioside B, a 6-*O*-substituted catalpol derivative, had higher antiinflammatory activity than catalpol did [9]. Scropoliosides contain one or more 6-*O*-substituted cinnamyl moieties, suggesting that the cinnamyl moiety is a critical structure that increases the antiinflammatory activity of catalpol derivatives. Ahmed *et al.* [19] also reported the antiinflammatory activity of scropolioside-D2 in a rat paw swelling experiment and thus concluded that compounds containing a cinnamyl moiety exhibit antiinflammatory activity. Similarly, other compounds with a cinnamyl moiety also exhibited an enhanced antiinflammatory activity [20,21,22]. In this study, scropoliosides with the cinnamyl moiety linked at different positions, namely C′′2-OH, C′′3-OH, and C′′4-OH, differently inhibited IL-1β, IL-8, and IFN-β mRNA expression, IL-1β secretion, and COX-2 activity (Table 1), demonstrating that the number and binding site of the cinnamyl moiety significantly affect antiinflammatory activity.

### 2.6. NF-κB Activity, Cytokine Expression and Release, and the Inhibitory Effect of Scropoliosides

Although all scropoliosides reduced NF-κB reporter activity to 40%–60%, their ability to inhibit IL-1β, IL-8, and IFN-β mRNA expression, IL-1β secretion, and COX-2 activity differed. Possible reasons are as follows: (1) In addition to NF-κB, the regulatory signals of IL-1β expression include C/EBPβ transcription factor and the p38/SAPK2 signaling pathway [23,24]; (2) IL-8 and IFN-β mRNA are expressed later than IL-1β mRNA and can be activated by the IL-1β autocrine loop (Figure 3). Several studies have reported that the transcriptional upregulation of IL-8 is mediated by IL-1β-stimulated activation of ERK1/2 and p38α MAPK pathway [25,26] and AP-1, ATF4, and NF-κB transcription factors [27,28]. Conversely, TLR4-induced IFN-β mRNA expression is regulated through JNK-, p38-, TIRAP-, and PI3K-dependent and MyD88-independent pathways and IRF-3, STAT1, and XBP-1 transcription factors [29,30,31,32]; (3) The maturation and secretion of IL-1β requires inflammasomes and other signals [9,33,34]. Overall, all scropoliosides, except scropolioside B, may not completely prevent these signals.

## 3. Experimental Section

### 3.1. General Information

The NMR spectra were recorded on a Bruker AM-400 spectrometer (Bruker, Billerica, MA, USA) at 400 MHz for ^1^H and 100 MHz for ^13^C in CD_3_OD. ESI-MS were obtained using a Thermo Finnigan LCQ Deca XP (Thermo Scientific, Waltham, MA, USA) equipped with an electrospray ionization source mass ion-trap. Silica gel (200 mesh to 300 mesh, Qingdao Haiyang Chemical Co., Ltd., Qingdao, China), C18 reversed-phase silica gel (150 to 200 mesh, Fuji Silysia Chemical, Ltd., Aichi, Japan), MCI gel (CHP20P, 75 μM to 150 μM, Mitsubishi Chemical Industries, Ltd., Tokyo, Japan), and Sephadex LH-20 gel (Pharmacia Biotech AB, Uppsala, Sweden) were used for column chromatography (CC). High-performance liquid chromatography was performed on an Angilent 1200 HPLC System (Angilent, Santa Clara, CA, USA) apparatus with an Eclipse XDB-C18 column (250 × 9.4 mm, 5 μm).

The activity of the luciferase reporter gene was assayed using dual-luciferase reporter 1000 assay system and detected using a Varioskan Flash microplate spectrophotometer (Thermo Scientific). Quantitative PCR was performed using a 7500 Fast Real-Time PCR System (Life Technologies, Grand Island, NE, USA) according to manufacturer instructions. The cells were lysed in ice-cold RIPA buffer and sonicated using a JY92-2D ultrasonic homogenizer (Ningbo Scientz Biotechnology Co., Ltd, Zhejiang, China). Lysates were pre-cleared through centrifugation at 12,000 *g* for 10 min at 4 °C. Aliquots of the cell lysate (50 or 100 µg of each sample) were resolved using SDS-PAGE and blotted onto a nitrocellulose membrane (Pall China, Shanghai, China). The optical density (OD) of each well of ELISA was measured immediately by using a SpectraMax 190 Absorbance Microplate Reader (Molecular Devices, Sunnyvale, CA, USA).

### 3.2. Cell Culture and Reagents

Human embryonic kidney 293 (HEK293) and human acute monocytic leukemia cell line THP-1 cells were purchased from the Chinese Academy of Sciences (Shanghai, China). The HEK293 cells were cultured in 100-mm tissue culture dishes containing Dulbecco’s modified Eagle’s medium with 10% newborn calf serum (Gibco, Life Technologies) at 37 °C in a humidified incubator in 5% CO_2_ and 95% air. The THP-1 cells were cultured in 100-mL flasks containing RPMI 1640 medium with 10% fetal bovine serum (Gibco) at 37 °C in a humidified incubator in 5% CO_2_ and 95% air. During experiments, the cells were plated in 24-well plates or 30-mm tissue culture dishes and incubated for 16 h for qPCR determination, or 24 h for ELISA and activity assay. All tested scropoliosides were dissolved in dimethyl sulfoxide (DMSO) that its final concentration in the culture medium was less than 0.2%.

### 3.3. Extraction and Isolation of Iridoid Glycosides from S. dentata Royle ex Benth

The isolation of scropoliosides A, B, and D was descibred in a previous study [35], which included the extraction and subsequent fractionation of the extract, evaporation of the *n*-butanol extract to dryness *in vacuo*, and silica-gel column chromatography elution of the resultant *n*-butanol fraction (572 g), using a gradient of EtOAc–EtOH (1:0–0:1) and finally EtOH to obtain fractions A–G. Fraction E was separated using MCI-gel column chromatography (MeOH–H_2_O, 20:80, 25:75, 30:70, 35:65, and 100:0) to obtain subfractions (scropoliosides A, B, and D, 6-*O*-α-l-(4′′-*O*-trans-*p*-coumaroyl) rhamnopyranosylcatalpol (named scropolioside F), and 6-*O*-α-l-(4′′-*O*-feruloyl) rhamnopyranosylcatalpol (named scropolioside G)). Fraction D2 was purified using Rp-18 (MeOH–H_2_O, 20:80–25:75 *v*/*v*) and sephadex LH-20 column shromatography (MeOH–H_2_O, 1:1 *v*/*v*) to obtain 6-*O*-methylcatapol (14 mg). Fraction D4 was isolated using Rp-18 column chromatography (MeOH–H_2_O, 20:80–50:50 *v*/*v*) and preparative HPLC on an Agilent Eclipse XDB-C18 column (5 μmol/L, 9.4 mm × 250 mm), followed by elution with CH3CN–H_2_O (18:82) to obtain scropoliosides F (44 mg) and G (19 mg). Using the same procedure, 6-*O*-α-l-(2′′-*O*-feruloyl) rhamnopyranosylcatalpol (named scropolioside H, 200 mg) and saccatoside (20 mg) were also obtained from fraction D4. Properties of scropolioside F, G, H, Saccatoside and 6-*O*-methylcatapol:

#### 3.3.1. 6-*O*-α-l-(4′′-*O*-trans-*p*-Coumaroyl)rhamnopyranosylcatalpol (Scropolioside F)

White amorphous powder. ESI-MS (pos.): 677 [M + Na]^+^, ESI-MS (neg.): 653 [M − H]^−^. ^1^H-NMR (400 MHz, CD_3_OD): δ_H_ 6.38 (2H, m, H-3/H-8′′′), 2.42 (1H, m, H-5), 4.03 (1H, d, *J* = 8.1 Hz, H-6), 3.66 (1H, br s, H-7), 2.57 (1H, dd, *J* = 8.3/8.9 Hz, H-9), 4.16 (1H, d, *J* = 13.1 Hz, H-10a), 3.82 (1H, d, *J* = 13.1 Hz, H-10b), 4.78 (1H, d, *J* = 7.9 Hz, H-1′), 3.41 (1H, t, *J* = 9.0 Hz, H-3′), 3.63 (1H, dd, *J* = 6.6/11.9 Hz, H-6′a), 5.00 (1H, br s, H-1′′), 1.17 (3H, d, *J* = 6.2 Hz, H-6′′), 7.48 (2H, d, *J* = 8.3 Hz, H-2′′′, 6′′′), 6.81 (3H, d, *J* = 8.3 Hz, H-3′′′′, 5′′′), 7.66 (1H, d, *J* = 15.9 Hz, H-7′′′), 5.05–5.12 (3H, m, H-1, H-4, H-4′′), 3.20-3.35 (4H, H-2′, 4′, 5′, 3′′), 3.88–3.94 (4H, H-6′b, 2′′, 5′′). ^13^C-NMR (100 MHz, CD_3_OD): δ_C_ 95.3 (C-1), 142.5 (C-3), 103.6 (C-4), 37.4 (C-5), 84.2 (C-6), 59.6 (C-7), 66.7 (C-8), 43.4 (C-9), 61.6 (C- l0), 99.8 (C-l′), 74.9 (C-2′), 77.8 (C-3′), 71.9 (C-4′), 78.7 (C-5′), 63.1 (C-6′), 100.6 (C-1"), 72.6 (C-2"), 70.4 (C-3"), 75.4 (C-4"), 68.4 (C-5"), 18.0 (C-6"), 127.3 (C-1′′′), 131.4 (2′′′ and C-6′′′), 117.0 (C-3′′′ and C-5′′′), 161.4 (C-4′′′), 147.1 (C-7′′′), 115.2 (C-8′′′), 169.1 (C-9′′′).

#### 3.3.2. 6-*O*-α-l-(4′′-*O*-Feruloyl)rhamnopyranosylcatalpol (Scropolioside G)

White amorphous powder. ESI-MS (pos.): 707 [M + Na]^+^, ESI-MS (neg.): 683 [M − H]^−^. ^1^H-NMR (400 MHz, CD_3_OD): δ_H_ 6.38 (1H, br d, *J* = 5.7 Hz, H-3), 2.42 (1H, m, H-5), 4.03 (1H, d, *J* = 8.1 Hz, H-6), 3.66 (1H, br s, H-7), 2.57 (1H, dd, *J* = 8.0/9.0 Hz, H-9), 4.16 (1H, d, *J* = 13.1 Hz, H-10a), 3.82 (1H, d, *J* = 13.1 Hz, H-10b), 4.78 (1H, d, *J* = 7.9 Hz, H-1′), 3.41 (1H, t, *J* = 9.0 Hz, H-3′), 3.63 (1H, dd, *J* = 6.6/11.8 Hz, H-6′a), 4.99 (1H, br s, H-1′′), 1.18 (3H, d, *J* = 6.2 Hz, H-6′′), 7.21 (1H, s, H-2′′′), 6.82 (1H, d, *J* = 8.0 Hz, H-5′′′), 7.10 (1H, d, *J* = 8.0 Hz, H-6′′′), 7.65 (1H, d, *J* = 15.9 Hz, H-7′′′), 6.41 (1H, d, *J* = 15.9 Hz, H-8′′′), 5.05–5.10 (3H, m, H-1, H-4, H-4′′), 3.20–3.35 (4H, H-2′, 4′, 5′, 3′′), 3.86-3.94 (4H, H-6′b 2′′, 5′′). 13C-NMR (100 MHz, CD_3_OD): δ_C_ 95.3 (C-1), 142.5 (C-3), 103.6 (C-4), 37.4 (C-5), 84.2 (C-6), 59.6 (C-7), 66.7 (C-8), 43.4 (C-9), 61.6 (C- l0), 99.8 (C-l′), 74.9 (C-2′), 77.8 (C-3′), 71.9 (C-4′), 78.7 (C-5′), 63.1 (C-6′), 100.6 (C-1"), 72.6 (C-2"), 70.4 (C-3"), 75.4 (C-4"), 68.4 (C-5"), 18.0 (C-6"), 127.9 (C-1′′′), 111.9 (C-2′′′), 150.8 (C-3′′′), 149.5 (C-4′′′), 115.6 (C-5′′′), 124.3 (C- 6′′′), 147.3 (C-7′′′), 116.6 (C-8′′′), 169.0 (C-9′′′), 56.6 (OMe).

#### 3.3.3. 6-*O*-α-l-(2′′-*O*-Feruloyl)rhamnopyranosylcatalpol (Scropolioside H)

White amorphous powder. ESI-MS (pos.): 707 [M + Na]^+^, ESI-MS (neg.): 683 [M − H]^−^. ^1^H-NMR (400 MHz, CD_3_OD): δH 6.39 (1H, dd, *J* = 6.0/1.7 Hz, H-3), 2.45 (1H, m, H-5), 4.04 (1H, d, *J* = 8.2 Hz, H-6), 3.67 (1H, br s, H-7), 2.59 (1H, dd, *J* = 7.8/8.9 Hz, H-9), 4.17 (1H, d, *J* = 13.1 Hz, H-10a), 3.82 (1H, d, *J* = 13.1 Hz, H-10b), 4.79 (1H, d, *J* = 7.9 Hz, H-1′), 3.42 (1H, t, *J* = 9.0 Hz, H-3′), 3.64 (1H, dd, *J* = 6.7/11.9 Hz, H-6′a), 5.05 (1H, d, *J* = 1.3 Hz, H-1′′), 5.17 (1H, dd, *J* = 3.4/1.3 Hz, H-2′′), 3.95 (1H, dd, *J* = 3.4/9.6 Hz, H-3′′), 3.51 (1H, t, *J* = 9.6 Hz, H-4′′), 3.77 (1H, dd, *J* = 9.6/6.2 Hz, H-5′′), 1.33 (3H, d, *J* = 6.2 Hz, H-6′′), 7.24 (1H, d, *J* = 1.5 Hz, H-2′′′), 6.84 (1H, d, *J* = 8.3 Hz, H-5′′′), 7.11 (1H, dd, *J* = 8.3/1.5 Hz, H-6′′′), 7.68 (1H, d, *J* = 15.9 Hz, H-7′′′), 6.46 (1H, d, *J* = 15.9 Hz, H-8′′′), 3.92 (3H, s, OMe); ^13^C-NMR (100 MHz, CD_3_OD): δ_C_ 95.3 (C-1), 142.4 (C-3), 103.6 (C-4), 37.4 (C-5), 84.5 (C-6), 59.6 (C-7), 66.7 (C-8), 43.4 (C-9), 61.6 (C- l0), 99.8 (C-l′), 75.0 (C-2′), 77.8 (C-3′), 71.9 ( C-4′), 78.7 (C-5′), 63.1 (C-6′), 97.8 (C-1"), 74.4 (C-2"), 70.7 (C-3"), 74.3 (C-4"), 70.4 (C-5"), 18.2 (C-6"), 127.9 (C-1′′′), 111.8 (C-2′′′), 150.9 (C-3′′′), 149.5 (C-4′′′), 115.4 (C-5′′′), 124.5 (C- 6′′′), 147.6 (C-7′′′), 116.6 (C-8′′′), 168.8 (C-9′′′), 56.6 (OMe).

#### 3.3.4. Saccatoside

White amorphous powder. ESI-MS (pos.): 661 [M + Na]^+^, ESI-MS (neg.): 637 [M − H]^−^. ^1^H-NMR (400 MHz, CD_3_OD): δ_H_ 6.38 (1H, dd, *J* = 6.0/1.7 Hz, H-3), 2.43 (1H, m, H-5), 4.03 (1H, d, *J* = 8.2 Hz, H-6), 3.65 (1H, br s, H-7), 2.57 (1H, dd, *J* = 7.7/9.7 Hz, H-9), 4.15 (1H, d, *J* = 13.1 Hz, H-10a), 3.80 (1H, d, *J* = 13.1 Hz, H-10b), 4.77 (1H, d, *J* = 7.9 Hz, H-1′), 3.40 (1H, t, *J* = 9.0 Hz, H-3′), 3.62 (1H, dd, *J* = 6.7/11.9 Hz, H-6′a), 5.02 (1H, d, *J* = 1.5 Hz, H-1′′), 5.17 (1H, dd, *J* = 3.5/1.5 Hz, H-2′′), 3.93 (1H, dd, *J* = 3.5/9.6 Hz, H-3′′), 3.49 (1H, t, *J* = 9.6 Hz, H-4′′), 3.75 (1H, dd, *J* = 9.6/6.2 Hz, H-5′′), 1.31 (3H, d, *J* = 6.2 Hz, H-6′′), 7.48 (2H, d, *J* = 8.6 Hz, H-2′′′, 6′′′), 6.81 (2H, d, *J* = 8.6 Hz, H-3′′′, 5′′′), 7.67 (1H, d, *J* = 15.9 Hz, H-7′′′), 6.40 (1H, d, *J* = 15.9 Hz, H-8′′′); ^13^C-NMR (100 MHz, CD_3_OD): δ_C_ 95.3 (C-1), 142.4 (C-3), 103.6 (C-4), 37.4 (C-5), 84.5 (C-6), 59.6 (C-7), 66.7 (C-8), 43.4 (C-9), 61.6 (C- l0), 99.8 (C-l′), 75.0 (C-2′), 77.8 (C-3′), 71.9 ( C-4′), 78.8 (C-5′), 63.1 (C-6′), 97.9 (C-1"), 74.4 (C-2"), 70.7 (C-3"), 74.3 (C-4"), 70.4 (C-5"), 18.2 (C-6"), 127.3 (C-1′′′), 131.4 (C-2′′′), 117.0 (C-3′′′), 161.5 (C-4′′′), 117.0 (C-5′′′), 131.4 (C- 6′′′), 147.3 (C-7′′′), 115.1(C-8′′′), 168.8 (C-9′′′).

#### 3.3.5. 6-*O*-Methylcatapol

White amorphous powder. ESI-MS (pos.): 399 [M + Na]^+^, ESI-MS (neg.): 435[M + CH_3_COO-]^−^. ^1^H-NMR (400 MHz, CD_3_OD): δ_H_ 5.47 (1H, d, *J* = 9.6 Hz, H-1), 6.45 (1H, dd, *J* = 1.8/6.0 Hz, H-3), 5.10 (1H, dd, *J* = 6.0/4.6 Hz, H-4), 2.68 (1H, m, H-5), 3.45 (1H, br d, *J* = 8.1 Hz, H-6), 3.71 (1H, br s, H-7), 2.78 (1H, dd, *J* = 7.8/9.6 Hz, H-9), 4.46 (1H, d, *J* = 13.1 Hz, H-10a), 4.51 (1H, d, *J* = 13.1 Hz, H-10b), 3.36 (3H, s, OCH3), 5.55 (1H, d, *J* = 7.9 Hz, H-1′), 4.15 (1H, dd, *J* = 7.9/8.9 Hz, H-2′), 4.30 (1H, t, *J* = 8.9 Hz, H-3′), 4.20 (1H, t, *J* = 8.9 Hz, H-4′), 4.04 (1H, ddd, *J* = 2.0/5.8/8.9 Hz, H-5′), 4.34 (1H, dd, *J* = 5.8/11.7 Hz, H-6′a), 4.57 (1H, dd, *J* = 2.0/11.7 Hz, H-6′b), 3.36 (3H, s, OCH_3_). ^13^C-NMR (100 MHz, CD_3_OD): δ_C_ 95.2 (C-1), 141.6 (C-3), 103.9 (C-4), 37.1 (C-5), 87.9 (C-6), 58.3 (C-7), 66.6 (C-8), 43.5 (C-9), 60.7 (C-l0), 100.5 (C-l′), 75.4 (C-2′), 79.4 (C-3′), 71.9 (C-4′), 78.7 (C-5′), 63.1 (C-6′), 57.8 (OMe).

### 3.4. Luciferase Assay

To assay NF-κB promoter activity, HEK-293 cells were transiently transfected with a luciferase reporter gene. pNF-κB-TA-Luc was purchased from Stratagene (La Jolla, CA USA). The cells were plated 1 day prior to transfection to obtain an approximately 80% confluence on the day of the transfection, when the DNA was diluted to 2 μg/100 μL of serum-free medium, and an appropriate amount of FuGENE HD transfection reagent (Promega, Madison, MI, USA) was added to achieve the optimal reagent-to-DNA ratio. The mixture was incubated for 0–15 min, and 100 μL of the mixture was added to each well for transfecting the cells. The cells were transfected for 5 h, and the mixture was then replaced with fresh media. One hour after the transfection, TNF-α was added to the cells, and the cells were incubated for 16 h. Luciferase activity was measured in the cell lysates using the Promega luciferase assay system according to the manufacturer’s instructions (Promega).

### 3.5. Quantitative Real-Time PCR

Total RNA was extracted using TRIzol reagent (Life Technologies) according to the manufacturer’s instructions. Real-time PCR amplification and detection were performed using the SYBR Green qPCR SuperMix-UDG with ROX (Life Technologies) in a fluorescence thermal cycler (StepOne real-time PCR system, Life Technologies) according to the manufacturer’s protocol. Relative mRNA expression levels were calculated by following the ΔΔCt method, using the following primers: GAPDH, IL-1β, IL-8, and IFN-γ (Table 2). All amplifications were conducted within the linear range of the assay and normalized to respective GAPDH levels by using SPSS Version 18.0 (SPSS Institute, Inc., Chicago, IL, USA).

**Table 2:** Primer sequences of the genes tested in this study.

Gene	Direction	Primer Sequences
IL-1β	Forward Reverse	5-AAACAGATGAAGTGCTCCTTCCAGG-3 5-TGGAGAACACCACTTGTTGCTCCA-3
IL-8	Forward Reverse	5-ATGGCTGCTGAACCAGTAGA-3 5-CTAGTCTTCGTTTTGAACAG-3
IFN-β	Forward Reverse	5-GCCTCAAGGACAGGATGAAC-3 5-AGCCAGGAGGTTCTCAACAA-3
GAPDH	Forward Reverse	5-AGAAGGCTGGGGCTCATTTG-3 5-AGGGGCCATCCACAGTCTTC-3

### 3.6. ELISA

Culture media from the control and treated cells were collected, centrifuged, and stored at −80 °C until further analysis. IL-1β was measured using the Abcam Human ELISA kit (Abcam, Cambridge, UK) according to the manufacturer’s instructions. IFN-β was measured using the Verikine Human IFN Beta ELISA kit (PBL Assay Science, Piscataway, NJ, USA) according to the manufacturer’s instructions. The standard or sample was added to each well, and the wells were incubated for 2.5 h at room temperature. The prepared biotin antibody was then added to each well, followed by incubation for 1 h at room temperature. Streptavidin solution was added, and the plates were incubated for 45 min at room temperature. Finally, TMB One-Step development solution was added to each well, and the plates were incubated for 30 min at room temperature. A stop solution was then added to each well, and the absorbance at 450 nm was recorded immediately.

### 3.7. Screening Assay for 15-Lipoxygenase Inhibitor

Lipoxygenase (LOX) inhibitory activity was measured using a Cayman lipoxygenase inhibitor screening assay kit (Cayman Chemical Company, Ann Arbor, MI, USA) according to the manufacturer’s instructions. The assay buffer was added to the blank and positive control wells and 15-LOX was added to the positive control wells, wells with 100% initial activity, and inhibitor wells. The solvent was added to the wells with 100% initial activity, and the inhibitor was added to the inhibitor wells. The substrate was then added to all the wells, and the wells were incubated for 5 min. Finally, the chromogen was added to each well, and the wells were incubated for 5 min to stop enzyme catalysis and develop the reaction. The absorbance at 490–500 nm was recorded.

### 3.8. COX-2 Activity Assay

COX-2 activity was measured using a Cayman COX activity assay kit (Cayman Chemical Company) according to the manufacturer’s instructions. The assay buffer, heme, standard, inhibitor, inactive sample, and sample were added to appropriate wells, and the wells were incubated for 5 min at 25 °C. The colorimetric substrate was then added to all of the wells. Finally, arachidonic acid solution was added to the wells, and the wells were incubated for 5 min at 25 °C. The absorbance at 590 nm was recorded.

### 3.9. Data Analysis

Each experiment was performed at least 3 times. The results were presented as means ± standard error of mean (SD). All data were analyzed using SPSS software, and a post hoc test in one-way ANOVA was used to determine the statistical significance of differences between the means. Differences were considered statistically significant when *p* < 0.05.

## 4. Conclusions

Our results show that scropoliosides differently inhibit the expression of various cytokines, IL-1β maturation and secretion, and COX-2 activity because of the different positions of linkage of the 6-*O*-substituted cinnamyl moieties, at C′′2-OH, C′′3-OH, or C′′4-OH. Moreover, the number of cinnamon moieties is closely related to the enhancement of anti-inflammatory activity. Among these compounds, scropolioside B has the strongest anti-inflammatory effects.

## Abbreviations

AGE: advanced glycation endproducts; COX-2: cyclooxygenase-2; DMSO: dimethyl sulfoxide; GAPDH: glyceraldehyde 3-phosphate dehydrogenase; HEK293: Human embryonic kidney 293 cell line; IFN-β: interferon-β; IL-1β: interleukin-1β; LPS: Lipopolysaccharides; LRR: Leucine-rich repeat protein; 15-LOX: 15-lipoxygenase; MCP-1: monocyte chemotactic protein-1; NACHT: neuronal apoptosis inhibitor protein; NF-κB: nuclear factor (NF)-κB; NLRP3: NACHT, LRR and PYD domains-containing protein 3; PGE_2_: prostaglandin E2; PYD: protein pyrin domain; THP-1 cell: Human acute monocytic leukemia cell line; TNF-α: tumor necrosis factor-α.
